# COVID19-induced immunosuppression and aggressive progression of primary cranial vault lymphoma presenting as a management challenge, a case report, and a literature review

**DOI:** 10.1186/s41983-022-00589-0

**Published:** 2022-12-14

**Authors:** Vikas Chandra Jha, Mohammad Shahnawaz Alam

**Affiliations:** Department of Neurosurgery, All India Institute of Medical Sciences, Patna, India

**Keywords:** Non-Hodgkin’s large cell, Lymphoma, Cranial vault, COVID-19, Mimicking meningioma, Case presentation

## Abstract

**Background:**

We needs to study Primary Large cell Non-Hodgkin’s Lymphoma of the cranial vault, which is rare, and its association with COVID19 has not been reported, which may have an immunosuppressive effect to aggravate its progression.

**Patient details:**

Our patient, a 53-year-old male, noticed fast growth of posterior cranial vault lesion from 2 to 10 cm size in last 6 months after COVID 19 affliction. MRI brain with contrast revealed lesions suggesting meningioma. The whole-body PET scan was normal. Following Subtotal excision of the mass, histopathology revealed large B-cell Non-Hodgkin’s lymphoma (DLBCL). Immunohistochemistry showed positive results for CD10, CD20, CD45 (LCA), ALK, and BCL-VE with a Ki-67 index of 90–95%. Following radiotherapy and chemotherapy patient is disease-free on imaging and doing well at 5 months of follow-up.

**Conclusions:**

Early intervention with excisional biopsy and timely chemo and radiotherapy in favorable immunostaining may add survival benefits even in malignant features induced by immunosuppressing diseases such as COVID19 in diffuse large B-cell lymphoma (DLBCL) of the scalp.

## Introduction

Primary cranial vault lymphoma is rare, and nearly 50 case reports are available mentioning it. Once it converts to a high-grade lesion, it presents as an osteolytic lesion, uncommonly as an osteoblastic lesion, and rarely with permeative transmission through the cortex [1]. Among these cases, concomitant calvaria and the epidural lesion are few and far between (5–6) [2, 3]. We did not find any study which could suggest its association with COVID 19, although it is commonly associated with other immunosuppressive conditions. We need to review the progression of the lesion, clinical behavior, management strategies, and prognosis in such scenario.

## Patient details

A 53 year male presented with multiple nodular huge scalp mass on the right side, which was noticed by the patient as a single lesion of grape size, 5 years back on the posterior cranial vault but in due course of the last 6 months, there was a progressive enlargement in size with an increase in lesions to 10 cm size. On the RT-PCR test, the patient had a history of COVID 19 positivity reported twice in the last 6 months. We did not find any enlarged lymph nodes or organomegaly on general examination. At that point, the clinical diagnosis was soft tissue sarcoma. On local examination, lesions were firm in consistency, nodular with an irregular nodular surface, with the largest scalp mass measuring about 10 cm in the right frontal and parietal region, which was painless and non-pulsating. The skin over the Swelling was normal, with patchy loss of hair, no tenderness, and no transparency or translucency. Neurological examination revealed no abnormalities, and the serological tests for human immunodeficiency virus, Epstein–Barr virus, and human T-cell lymphotropic virus were negative, and before surgery, he was COVID 19 RT-PCR test negative.

**Fig. 1:**
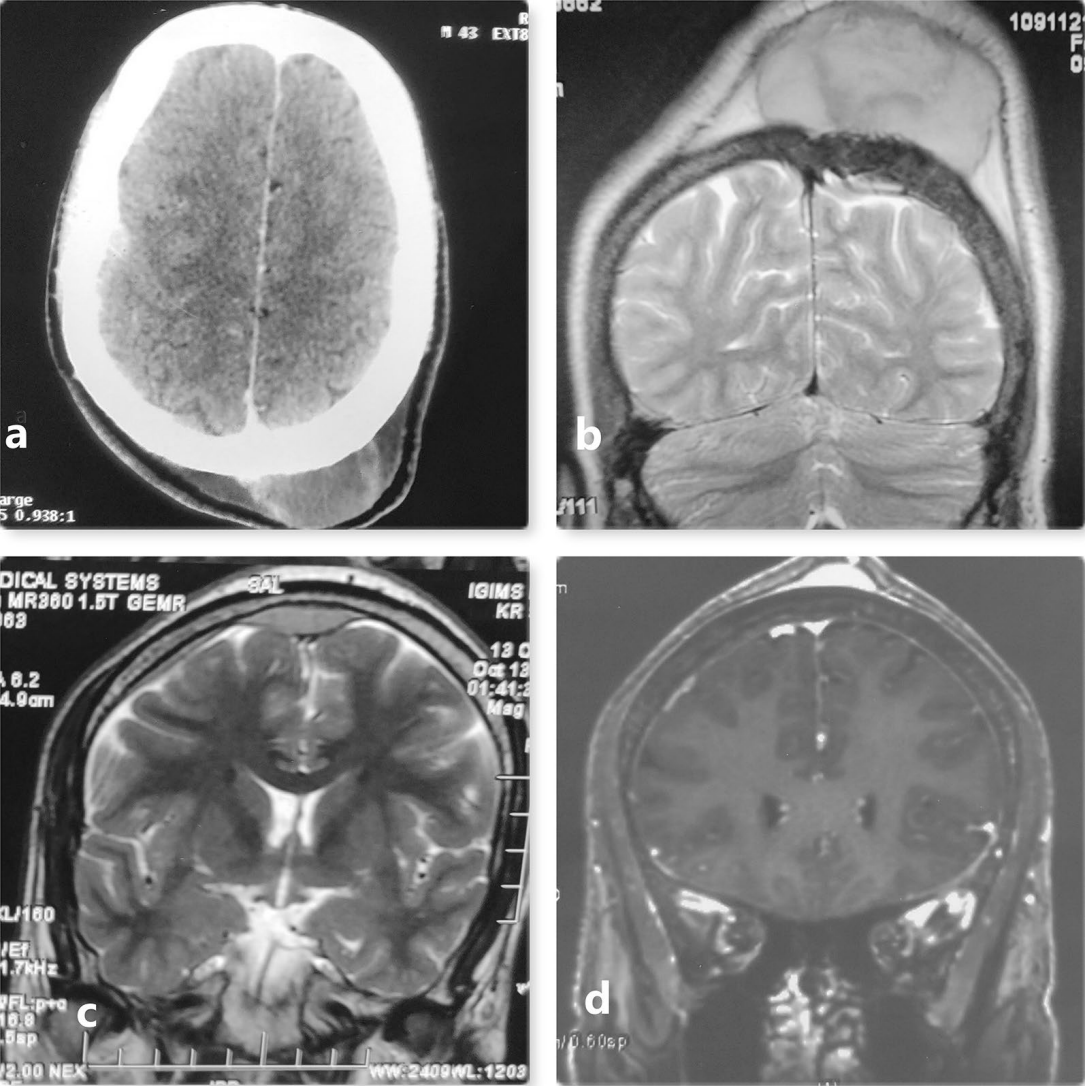
**a** Nonenhanced computerised tomography scan suggesting lesion of size 3 cm without underlying cortical changes in calvaria. lesion is isodense. **b** On Coronal T2Magnetic resonance imaging (MRI) images done 6 months later shows increase in size of the lesion to around 10 cm with new lesion in with cortical changes in underlying bone suggesting selective permeation of tumour tissue. **c** Coronal T2 weighted image suggestive of lesion in midline with involvement of anterior sagittal sinus and left parietal region Dural involvement. **d** Coronal contrast MRI suggesting homogenous contrast enhancement of the lesion with Dural tail sign present

**Fig. 2:**
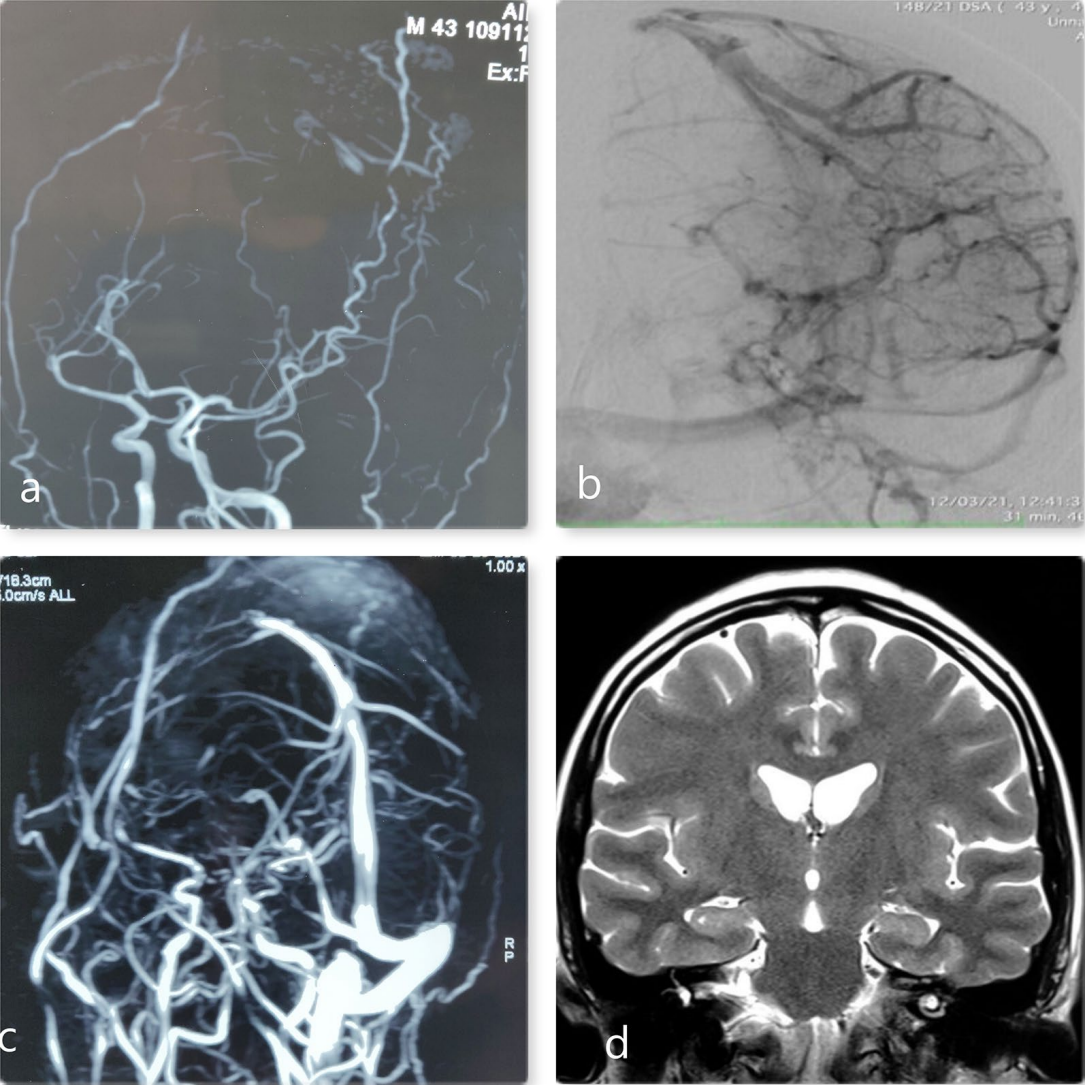
**a** MR arteriography suggestive of tumour feeding artery from both External carotid artery. **b** Digital subtraction angiography suggestive of tumour Blush present suggesting vascular tumour. **c** MR angiography suggestive of no visualisation of anterior sagittal sinus and left transverse sinus. **d** Postoperative image shows no residual lesion on Coronal T2 weighted images following radiotherapy and chemotherapy

Brain Non contrast computed tomographic (NCCT) examination showed Swelling (soft tissue opacity) in the right frontoparietal region with the exclusion of the skull invasion (Fig. 1a). No concurrent nodal or intracranial (extradural/meningeal) involvement or orbit invasion was noticed 6 months back. Brain Magnetic resonance imaging (MRI) was done 6 months after CT demonstrated a multiple-nodular huge scalp mass with heterogeneous signals which was isointense on T1 weighted imaging and heterogeneous signals on T2-weighted imaging (Fig. 1b, c). On T2 weighted images, there were hypointense changes in cortical bone, and patchy contrast enhancement suggested tumor infiltration (Fig. 1b). There were lesions in the anterior frontal region appearing to be epidural and compressing anterior sagittal sinus and a small epidural deposit on the left parietal region (Fig. 1c). On the Contrast scan, the lesion was homogenously contrast-enhancing with Dural tail positive (Fig. 1d), and digital subtraction angiography revealed a tumor supplied by bilateral external carotid artery (ECA) (Fig. 2a) with tumor blush present on DSA (Fig. 2b). Findings suggest calvaria atypical meningioma and non-visualization of the anterior sagittal sinus and left transverse sinus (Fig. 2c). PET scan whole body failed to identify any other evidence of primary lesion or systemic lymphoma/malignancy.

**Fig. 3:**
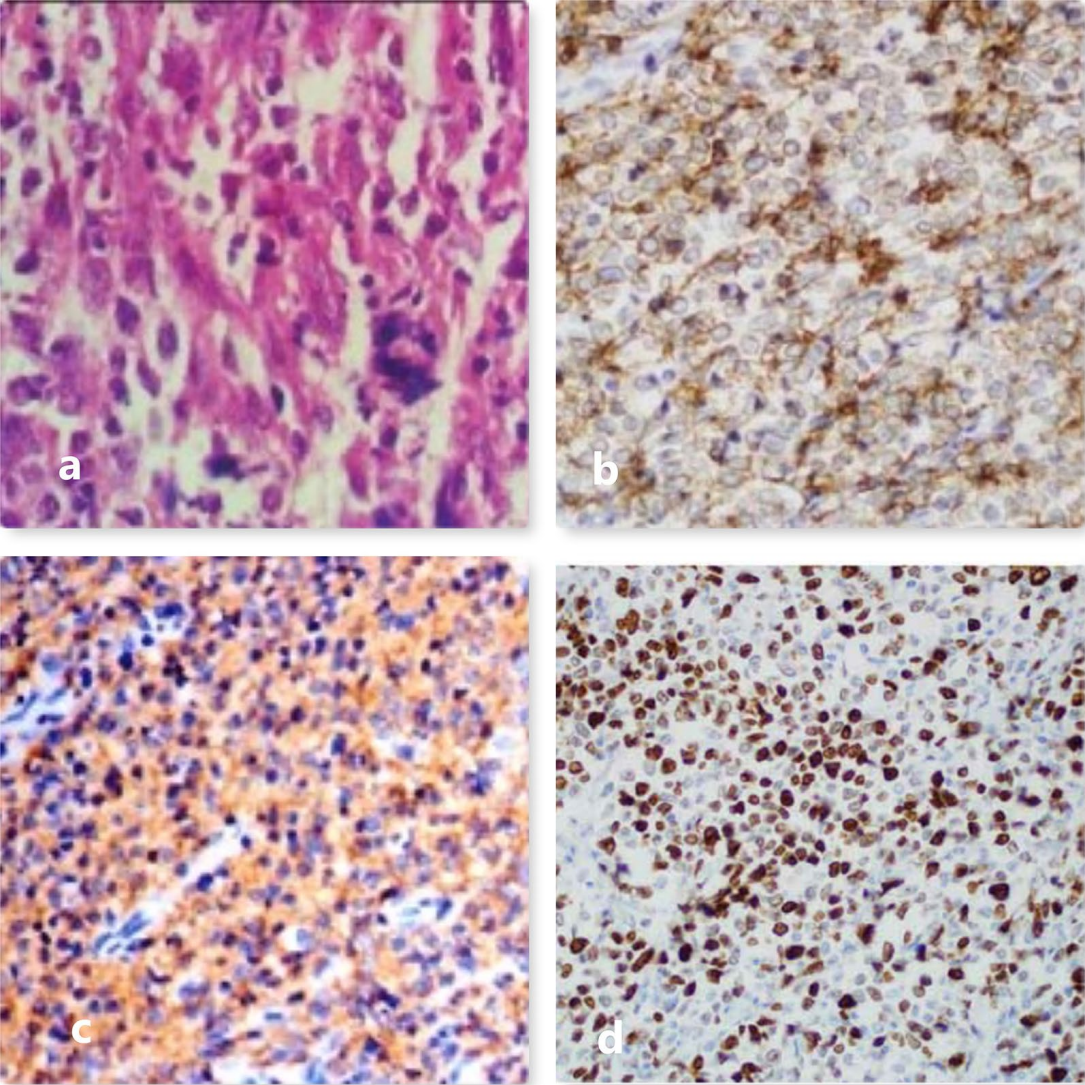
**a** Histopathology findings suggestive of Diffuse Large B cell Non-Hodgkin’s Lymphoma. **b** On immunostaining lesion was CD20 positive. **c** On immunostaining lesion was CD45 positive. **d** On immunostaining KI-67 marker shows proliferative index of 90–95%

We treated the patient with gross total excision of the large right parietal calvaria mass and curettage of the underlying bone. Gross total resection of the tumor was performed without excising small Dural deposits in the anterior frontal and left parietal region. We found the tumor-infiltrating galea, frontalis, and occipitalis muscles. It was firm to hard in consistency, mildly vascular, and yellowish. Microscopic examination of the tissue showed discohesive malignant cells arranged in sheets infiltrated by neutrophils and lymphocytes. Cells were large with marked pleomorphism, a high N:C ratio with prominent nucleoli, and scant cytoplasm. There were extensive areas of necrosis with frequent atypical mitotic figures. The pathological diagnosis was diffuse large B-cell NHL (DLBCL) (Fig. 3a). Immunohistochemistry showed positive results for CD10, CD20, CD45 (LCA), and ALK with a Ki-67 index of 90–95% (Fig. 3b, c). It is followed by cycles of chemotherapy with injection cyclophosphamide, doxorubicin, vincristine, and tab. prednisolone. (CHOP regimen) showed a good response. Local adjuvant External beam Radiotherapy of − 40 Gy @ − 2 Gy per fraction infraction for − 8 weeks was given to the right scalp region with a CHOP regimen. The patient has no residual lesion on repeat MRI at 5 month follow-up and doing well (Fig. 2d).

## Discussion

Non-Hodgkin’s lymphoma of the cranial cavity may be present due to metastatic deposit of systemic lymphoma in 1–6% cases and cranial vault in less than 1% cases, but primary NHL of the cranial vault is rare. [1, 2] Not more than 50 cases have been reported to date. Primary cranial vault lymphoma had been defined as localized lymphoma not involving other than the primary site for more than 6 months, once we made the initial diagnosis [3]. In primary calvaria lymphoma, the most common histopathology reported is NHL large B-cell type (39–78%), and in primary Dural lymphoma most common pathology reported is marginal B cell lymphoma which is low grade.

In the El Asri et al. meta-analysis, the average age at presentation was 60 years without any gender prevalence. The average duration was the onset of symptoms was average of 1 year [4]. In the present case report, the patient noticed swelling 5 years back on the right parieto-occipital region, which was of the size of grapes, but he was inflicted with COVID twice within 1 year at an interval of 6 months, and there was a fast progression of size and patient noticed another three lesions on the scalp. We may attribute this fast progression to the immunosuppression caused by COVID 19 treatment and the immunosuppressive therapy he had received during his treatment, as he developed a drastic fall in oxygen saturation of 80% in both episodes of COVID 19 infection. In studies of El Asri et al. and Ferreri et al., the immunosuppressed condition was reported in nearly 11% of patients [4, 5].

In the present study, tumor characteristics on T1 and T2 weighted MRI were almost similar to the other reported patients with similar histopathology, and it homogenously contrasts enhanced with Dural tail sign. We noticed selective permeation of tumor mass through the cranial vault with tumor deposit on the inner table of calvaria and seeding of the dura in the anterior frontal region, causing mass effect and no visualization of the anterior sagittal sinus. On DSA tumor was being fed from bilateral ECA with tumor blush present suggesting vascular lesion, and left transverse sinus was also not visualized. Angiographic findings favor meningioma as lymphomas are not reported to have vascular blush on angiography except in 2–3 reported patients in the literature [3, 6].

Our patient’s MRI findings did not reveal any widening of diploic space, and there were slightly cortical changes and patchy contrast enhancement suggestive of permeation of calvaria cortex by malignant lymphomatous cells. Anterior sagittal sinus was not visualized, probably due to overlying compression by tumor mass and metastatic deposit through emissary’s veins. As the lesion was high-grade permeation of left transverse sinus through emissary veinous channel leading to thrombosis may be the reason. Such selective permeation of malignant lymphomatous cells was reported in the study by Ochiai et al. and Galarza et al. [7, 8]. In the study by Kissling et al., the lymphomatous mass resembling meningioma has diffuse osteolytic features with thrombosis of the posterior third sagittal sinus [3].

Such atypical radiological findings probably have not been reported.

Faster progression of the tumor was reflected by high KI-67 proliferation index, but one good prognostic feature was the absence of BCL-2 staining, which helps in good chemotherapy and radiotherapy response as reported in different studies [9]. In the previous, reported cases mimicking meningioma BCL-2 staining in FISH were presented, suggesting a poor response to treatment [3, 8]. We did not find an extended follow-up in these reports. In the study by Ciarpaglini et al., high-grade lesions survive from 10 to 17 months, and in low-grade 5 years, survival is around 40–50% [10]. Although our patient developed a multicentric tumor over time, less tumor bulk and good histopathology for chemo and radiotherapy had given the advantage of better survival. He is asymptomatic after treatment on 5 months of follow-up and no fresh lesion on repeat MRI.

We thought to report this case due to unusual association with COVID 19 and atypical presentation, which was not reported in the earlier reported case, presenting a diagnostic and management dilemma to us. Patients having cranial vault primary DLBCL with the benign course may convert into aggressive lesions following infliction with the immunosuppressed state as in the present patient study and may present as a diagnostic dilemma. However, early excisional diagnosis and favorable immunostaining with BCL-2 may add survival benefits.

## Conclusions

Cranial vault primary lymphoma as DLBCL may have slow progression, but it may acquire aggressive features induced by immunosuppressing infection and medication and present a challenge in diagnosing and management. Early intervention with excisional biopsy and timely chemo and radiotherapy in favorable immunostaining may add survival benefits even in the presence of malignant features.

## Data Availability

It will be provided whenever required.
